# Five-Year Survivorship of Total Hip Arthroplasty With a Proximally Coated, Medially Collared, Triple-Tapered Femoral Stem: A Retrospective, Multicenter Registry Review

**DOI:** 10.7759/cureus.59462

**Published:** 2024-05-01

**Authors:** Michael Hunter, David W Fawley, Rodrigo Diaz, William Barrett, Sean Croker, Robert Gorab

**Affiliations:** 1 Orthopedics, Hoag Orthopedic Institute, Irvine, USA; 2 Clinical Research, DePuy Synthes, Warsaw, USA; 3 Orthopedics, Proliance Orthopedic Associates, Renton, USA; 4 Orthopedic Surgery, Hoag Orthopedics Institute, Irvine, USA

**Keywords:** total hip arthroplasty revision, periprosthetic fracture, uncemented tha, implant survivorship, total hip arthroplasty (tha)

## Abstract

Background

The anterior approach for total hip arthroplasty (THA) has gained popularity in recent years. Some surgeons have been hesitant to adopt the approach due to concerns over increased complications such as intraoperative fracture, stem loosening, and stem revision. This study aims to evaluate the all-cause revision rate and survivorship of a collared, triple-tapered stem that was designed specifically for use with the anterior approach in THA to enhance outcomes and reduce adverse events.

Methodology

A retrospective outcomes review was conducted to assess survivorship and clinical outcomes for a specific proximally coated, medially collared triple-tapered (MCTT) femoral stem.

Results

In a cohort of 5,264 hips, Kaplan-Meier survivorship estimates (95% confidence interval [CI]; *N* with further follow-up), with survivorship defined as no revision of any component for any reason at five years after the index procedure, were 98.9% (97.8%-99.4%; 43) under the clinical assumption and 99.6% (99.4%-99.7%; 894) under the registry assumption. With survivorship defined as stem revision for any reason, survivorship estimates at five years postoperatively were 99.6% (99.3%-99.8%; 43) under the clinical assumption and 99.8% (99.7%-99.9%; 894) under the registry assumption. The mean follow-up time was 94.52 days (standard deviation [SD] 2.24, range 90.03-96.02). At five years postoperatively, the mean Harris Hip Score was 95.19, and the mean Hip Disability and Osteoarthritis Outcome Score Junior (HOOS JR) score was 98.66.

Conclusions

Our evaluation demonstrates excellent construct and stem survivorship and very low complication rates at midterm postoperative follow-up.

## Introduction

Total hip arthroplasty (THA) has become one of the most frequent and successful procedures performed in the modern surgical era. The number of THA procedures performed each year continues to rise and is expected to increase to almost 600,000 by the year 2030 [1]. Several factors have contributed to the continued success of THA. These include improved bearing surfaces, novel femoral stem designs, optimized peri-operative pain management, and recent advances in surgical approaches. The posterior and direct lateral approaches for THA have been commonplace historically, but due to concern over complications such as abductor weakness and dislocation risk, as well as enhanced interest in rapid recovery pathways, newer surgical techniques have been explored in recent years [2]. Over the past decade, there has been a significant shift toward less invasive surgical approaches, such as the direct anterior approach (DAA) and mini-posterior approach, in an attempt to improve early mobility and decrease peri-operative pain [3]. The DAA is a tissue-sparing approach for THA and has been gaining popularity due to reports of a quicker functional recovery, lower dislocation risk, more accurate placement of components, and restoration of leg length and offset [4-9]. Despite the advantages reported with the DAA, it has been called into question for its steep learning curve and concern over increased intraoperative complication risks [10]. Several studies have described a higher rate of wound complications, component malposition, intraoperative fracture, decreased visualization, and neurovascular injury [11-14]. Follow-up studies have reported on the efficacy of the direct anterior hip approach by surgeons who are well-versed in the technique and aware of its potential pitfalls that can increase complication rates [15]. Specifically, regarding periprosthetic fracture risk, Berend et al. reported an early periprosthetic fracture rate of 0.9% utilizing a single taper stem with a trend toward increased rates among elderly women [16,17]. The risk of intraoperative and early postoperative fracture was 1.4% with single wedge taper designs as compared to 0% in Christensen’s series [18]. Barnett et al. reported a 0.84% proximal femur fracture rate and 0.23% dislocation rate during the first 90 days of the procedure, comparable to other manuscripts regardless of the surgical approach utilized [19]. This further confirms that complications can be minimized by surgeons familiar with the approach.

To mitigate the complication risks and optimize long-term femoral fixation with anterior hip surgery, new femoral stem designs and associated instrumentation have been developed in recent years. In 2016, an FDA-cleared hydroxyapatite-coated, medially collared triple-tapered (MCTT) hip stem was released, designed specifically for less invasive approaches to THA, such as the DAA. Its design rationale was centered around providing optimized initial implant stability and early bone ingrowth when using less invasive approaches. Chitnis et al. reported on their results using the MCTT stem in 1200 patients, extracted from a retrospective database. They found a lower revision rate at 3 years as compared to conventional hip stems (1.03% vs. 2.63%) [20]. The recent American Joint Replacement Registry (AJRR) data from 2020 also showed that the MCTT stem had the lowest revision rate at three years among all stems analyzed [21]. However, given the retrospective nature of these studies, a more robust analysis of a multicentered database is required to prospectively evaluate the success of this novel hip stem. This study aimed to determine the early complication profile and mid-term survivorship and clinical outcomes of the MCTT stem by aggregating data from several high-volume centers that exclusively utilize less invasive approaches to THA.

## Materials and methods

A retrospective outcomes review was conducted using data collected from an ongoing, prospective, standard-of-care, multicenter registry. Informed consent was obtained for all patients before participation, and Institutional Review Board (IRB) review and approval were obtained for the registry and all participating sites. All patients who received an MCTT stem (ACTIS stem with a PINNACLE® Hip Solutions cup, DePuy Synthes, Warsaw, IN) were included. Preoperative and postoperative clinical assessments included the Harris Hip Score, Hip Disability and Osteoarthritis Outcome Score Junior (HOOS JR) score, and a registry-specific hip evaluation questionnaire [22]. The procedures followed were per the Western Institutional Review Board Copernicus Group (WCG IRB, study number 1166116).

Statistical analysis

Clinical assessments were summarized with sample size, mean, and standard deviation (SD) for numeric scores and with sample size and percentages for categorical responses. It is recognized that sites within the registry have different standards of care regarding clinical follow-up visits. Therefore, standardized registry visit windows were established. If an individual patient had multiple visits within the standardized window, then only the latest visit was included in the summaries.

**Figure 1:**
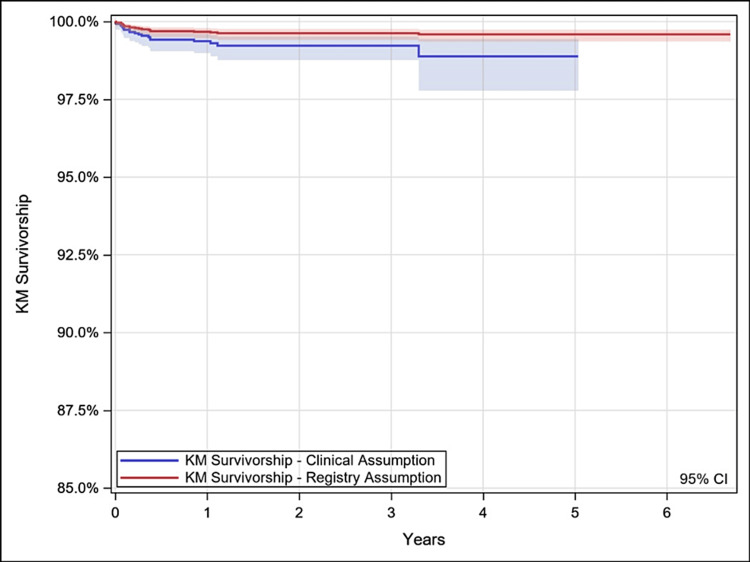
Kaplan-Meier (KM) survivorship estimates over time (any component, any reason). KM survivorship = % of stem survivorship. Years = Years since index surgery (*n*). CI, confidence interval

**Figure 2:**
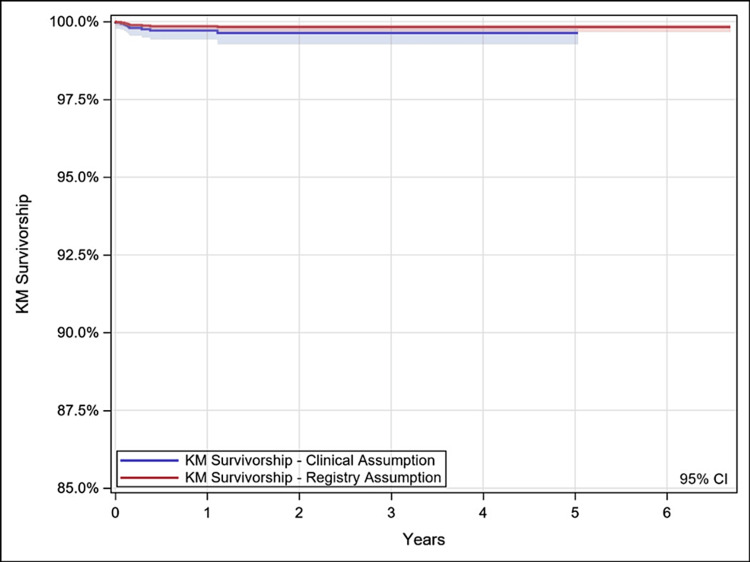
Kaplan-Meier (KM) survivorship estimates over time (stem revision). KM survivorship = % of stem survivorship. Years = Years since index surgery (*n*). CI, confidence interval

Kaplan-Meier survivorship was performed with revision of the stem and revision of any component as endpoints. For each endpoint, two survivorship analyses were performed with differing censoring assumptions. First, unrevised patients were censored at the last clinical follow-up [clinical assumption (CA)], making no assumptions on the implant survivorship beyond the patient’s last visit. Second, unrevised patients were censored at the date of database extract [registry assumption (RA)], assuming that if a revision or death has not been reported then the devices remain implanted. In an observational registry data setting it is believed that RA tends to overestimate survivorship estimates, whereas CA has the potential to underestimate survivorship; this report included both analysis methods to improve the transparency of the data analysis and can be seen in Figures 1-2. In all cases, survival estimates were truncated at 40 hips remaining at risk. For the survivorship of the stem component, patients were censored at the time of removal of other components.

A total of 5,264 MCTT stems were implanted in cementless primary THA between February 2016 and January 2023. Two thousand four hundred eighty-four (47.2%) of the enrolled hips were women. The mean age was 64.7 years (SD 10.20, range 21-93) and the mean BMI was 28.2 (SD 5.13, range 15-74). Most primary diagnoses were osteoarthritis (4,696, 89.2%). The surgical approach was direct anterior for 4,676 (88.8%) hips, anterolateral/Hardinge for 402 (7.6%) hips, and other approaches for 183 (3.5%) hips.

## Results

There were 20 revisions (any component for any reason), 17 of which occurred before one year postoperatively, and 9 of 20 involving the femoral stem. All-cause revisions were performed for infection (12), dislocation (6), liner dissociation (1), and bone fracture (1). Additional details for reported revisions are shown in Table 1. Fifteen perioperative medical and surgical complications were reported and included: renal/urinary events (4), cardiac events (4), respiratory events (2), foot drop (2), periprosthetic fracture treated with Open Reduction and Internal Fixation (ORIF) (1), unicortical lateral femoral fracture (1), and severe cutaneous allergy from wound dressing (1). A Vancouver Type B2 fracture was reported one month post-surgery and treated with ORIF. A case of heterotopic ossification requiring excision was reported one year post-surgery.

**Table 1:** Revision details. x = component revised.

Revision reason	Date of surgery	Date of revision*	Time to revision (Days)	Stem revised	Cup revised	Head revised	Liner revised
Bone fracture treated with cable	Nov 20, 2018	Apr 1, 2019	132	-	-	-	x
Dislocation	Oct 23, 2019	Aug 31, 2020	313	-	-	x	x
Dislocation	Sep 19, 2018	Oct 2, 2019	378	-	-	x	x
Dislocation	Sep 9, 2019	Sep 12, 2019	3	-	-	x	x
Dislocation	Jun 15, 2017	Oct 2, 2020	1,205	-	-	x	x
Dislocation	Nov 4, 2020	Nov 4, 2020	0	x	-	-	x
Dislocation	Oct 11, 2019	Nov 13, 2019	33	-	x	x	x
Infection	Feb 13, 2018	Mar 13, 2018	28	-	-	x	x
Infection	Aug 27, 2020	Nov 13, 2020	78	-	-	x	x
Infection	Nov 19, 2019	Mar 3, 2020	105	x	x	x	x
Infection	Oct 18, 2016	Nov 28, 2017	406	x	x	x	x
Infection	Aug 8, 2017	Sep 20, 2017	43	x	x	x	x
Infection	Jul 27, 2017	Dec 12, 2017	138	-	-	x	x
Infection	May 14, 2019	Jun 18, 2019	35	x	x	x	x
Infection	May 6, 2019	Jun 27, 2019	52	x	x	x	x
Infection	Feb 16, 2021	Apr 13, 2021	56	x	x	x	x
Infection	Apr 9, 2018	May 1, 2018	22	x	-	-	x
Infection	Dec 13, 2017	May 1, 2018	139	x	x	x	x
Infection	Feb 28, 2022	Apr 26, 2022	57	-	-	x	x
Liner dissociation	Mar 2, 2017	Jun 3, 2017	93	-	-	x	x

Survivorship

**Figure 3:**
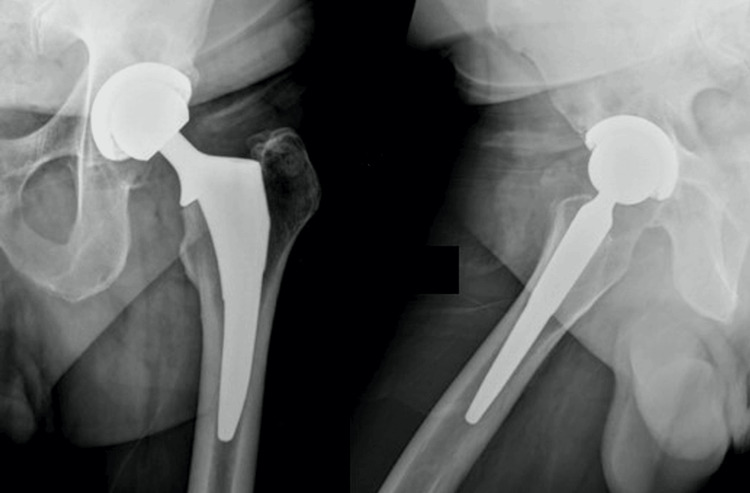
Anterior/posterior and lateral images of medial collared triple-tapered stem implanted via a direct anterior approach. X-ray images of medially collared triple-tapered (MCTT) stem at six weeks post-implantation.

**Figure 4:**
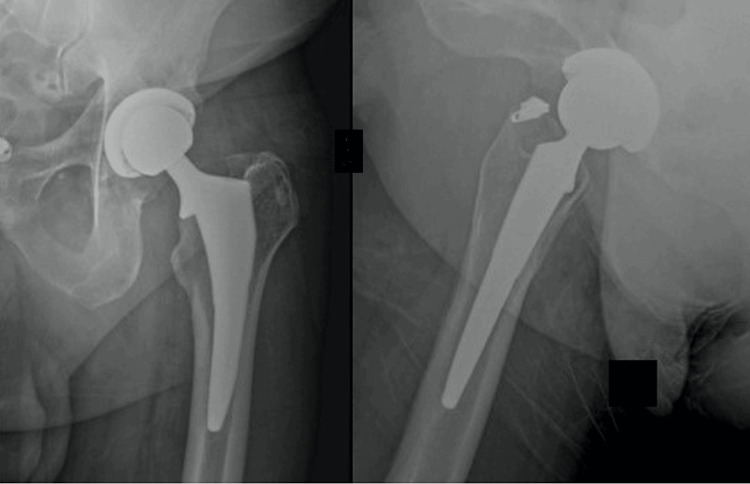
Anterior/posterior and lateral images of medially collared triple-tapered stem implanted via a direct anterior approach. X-ray images of medially collared triple-tapered (MCTT) stem at seven years post-implantation.

Kaplan-Meier survivorship estimates (95% confidence interval [CI]; *N* with further follow-up), with survivorship defined as no revision of any component for any reason, were 98.9% (97.8%-99.4%; 43) at five years under the clinical assumption and 99.6% (99.4%-99.7%; 894) under the registry assumption. Additional details are provided in Table 2 and Figures 3-4. With survivorship defined as stem revision for any reason, estimates were 99.6% (99.3%-99.8%; 43) at five years under the clinical assumption and 99.8% (99.7%-99.9%; 894) under the registry assumption. Additional details are provided in Table 3 and Figure 4.

**Table 2:** Kaplan-Meier survivorship estimates (any component, any reason). CI, confidence interval; CA, clinical assumption; RA, registry assumption

Any component, any reason	0 year	1 year	2 years	3 years	4 years	5 years
Hips revised (*n*)	0	17	19	19	20	20
Hips remaining (CA) (*n*)	5,264	1,736	800	364	189	43
Hips remaining (RA) (*n*)	5,264	4,687	3,896	2,965	1,904	894
CA survival estimate (95% CI) (%)	100.0 (100.0-100.0)	99.4 (99.0-99.6)	99.2 (98.8-99.5)	99.2 (98.8-99.5)	98.9 (97.8-99.4)	98.9 (97.8-99.4)
RA survival estimate (95% CI) (%)	100.0 (100.0-100.0)	99.7 (99.5-99.8)	99.6 (99.4-99.8)	99.6 (99.4-99.8)	99.6 (99.4-99.7)	99.6 (99.4-99.7)

**Table 3:** Kaplan-Meier survivorship estimates (femoral component, any reason). CA, clinical assumption; RA, registry assumption

Femoral component, any reason	0 year	1 year	2 years	3 years	4 years	5 years
Stems revised (*n*)	0	8	9	9	9	9
Hips remaining (CA) (*n*)	5,264	1,736	800	364	189	43
Hips remaining (RA) (*n*)	5,264	4,687	3,896	2,965	1,904	894
CA survival estimate (95% CI) (%)	100.0 (100.0-100.0)	99.7 (99.4-99.9)	99.6 (99.3-99.8)	99.6 (99.3-99.8)	99.6 (99.3-99.8)	99.6 (99.3-99.8)
RA survival estimate (95% CI) (%)	100.0 (100.0-100.0)	99.8 (99.7-99.9)	99.8 (99.7-99.9)	99.8 (99.7-99.9)	99.8 (99.7-99.9)	99.8 (99.7-99.9)

Clinical outcomes

The mean total Harris Hip Scores (SD; *N*) were 53.26 (13.86; 4,243), 95.56 (7.93; 799), and 95.19 (6.72; 64) at pre-op, two years, and five years post-op, respectively. Mean HOOS JR total scores (SD; *N*) were 51.31 (15.96; 1,031), 95.93 (10.37; 355), and 98.66 (4.34; 22) at pre-op, two years, and five years post-op, respectively. Additional details, including Harris Hip activities, pain, and function scores are included in Table 4. Thigh pain was reported in 6% of hips at one year and 4.8% of hips at five years postoperatively. Additional details regarding patient-reported thigh pain are presented in Table 5.

**Table 4:** Clinical outcomes. The data are represented as *N* (total number of subjects), SD (standard deviation), and mean. HOOS JR, Hip Disability and Osteoarthritis Outcome Score Junior

	Preoperative	<1 year [1-303 days]	1 year [304-668 days]	2 years [669-1034 days]	3 years [1035-1399 days]	4 years [1400-1764 days]	5 years [1765-2737 days]	
Harris Hip Total Score								
Mean	53.26	90.03	95.66	95.56	94.70	96.02	95.19	
SD	13.86	11.50	7.56	7.93	9.19	5.61	6.72	
N	4,243	1,651	1,952	799	206	195	64	
Harris Hip Activities Score								
Mean	9.24	12.23	12.91	12.69	12.46	12.41	12.44	
SD	2.60	1.94	1.50	1.66	1.59	1.49	1.55	
N	4,260	1,659	1,962	804	208	196	64	
Harris Hip Pain Score								
Mean	14.92	39.76	42.19	42.34	41.79	42.67	43.09	
SD	8.48	6.88	5.01	5.28	6.27	3.99	3.08	
N	4,296	1,693	1,972	807	209	197	64	
Harris Hip Function Score								
Mean	20.82	29.32	31.72	31.69	31.28	31.96	30.83	
SD	6.85	5.20	3.11	3.38	3.79	2.37	4.32	
N	4,286	1,671	1,960	803	209	196	64	
HOOS JR Score								
Mean	51.31	85.49	96.00	95.93	97.22	99.07	98.66	
SD	15.96	14.03	9.72	10.37	7.29	4.60	4.34	
N	1.031	853	733	355	102	118	22	

**Table 5:** Thigh pain.

	Preoperative	<1 year [1-303 days]	1 year [304-668 days]	2 years [669-1034 days]	3 years [1035-1399 days]	4 years [1400-1764 days]	5 years [1765-2737 days]
None (*n*)	564	787	1,525	604	184	182	59
Slight (*n*)	375	151	38	7	4	3	0
Mild (*n*)	600	108	33	16	3	0	2
Moderate (*n*)	928	75	21	10	6	3	1
Marked (*n*)	602	6	2	0	3	0	0
Completely disabled (*n*)	26	1	3	0	0	0	0
*N*, Total (*n*)	3,095	1,128	1,622	637	200	188	62
Any thigh pain (%)	81.8%	30.2%	6.0%	5.2%	8.0%	3.2%	4.8%

## Discussion

During DAA THA, femoral exposure and broaching can be a source of angst for surgeons, most commonly occurring during the learning curve of the technique. Suboptimal femoral exposure can lead to the malpositioning of components and a less-than-ideal femoral broach envelope, with the potential consequence of femoral stem loosening or femoral fracture [23]. This can be further accentuated by longer, straight stem designs that do not permit easy access to the femoral canal. Panichkul et al. reported a higher rate of wedge taper stem revision utilizing the anterior approach as opposed to the direct lateral and posterior approach [24]. A prior study from one of our institutions also revealed that the rate of aseptic loosening was higher in taper wedge stems as opposed to a collared, dual taper stem in the DAA [25]. The Dutch registry illuminated an association between early femoral loosening when using an implant with a large lateral shoulder versus an anatomic shoulder [26]. These concerns directed interest in a more appropriate stem design to optimize outcomes in less invasive approaches to THA. In a landmark paper by Noble et al. classifying femoral geometry in relationship to stem design, it was apparent that endosteal geometry should be matched closely for optimal bony support and was reaffirmed in a recent paper by Warth et al. [27,28]. Also, given the nature of varied femoral anatomy, multiple neck lengths were needed to accurately restore patient anatomy.

As noted in the Introduction section, the MCTT stem was released as an FDA-cleared, triple taper, hydroxyapatite-coated, medially collared hip stem designed specifically for less invasive approaches to THA, such as the DAA. Its design rationale was centered around providing optimized initial stability and early bone ingrowth when using a less invasive approach. Recent studies have shown that collared stems have a lower periprosthetic fracture risk and greater initial implant stability than similar geometry uncollared stems [29,30,31]. To improve initial implant stability, enhanced design features of this triple taper geometry also include incremental growth in the anterior/posterior plane by 1 mm per size, as well as a 1/4 degree increase in taper angle per size. The lateral shoulder of the implant was reduced to promote ease of insertion and avoid damage to the external rotators and abductors during anterior hip surgery. A beveled lateral distal tip was also designed to decrease femoral canal perforation and allow for easy insertion across multiple femoral geometries. Many of the features of this stem design are unique and aimed to decrease intraoperative and postoperative complications associated with the anterior approach and were not seen in prior stem designs.

This registry evaluation of the MCTT stem shows excellent survivorship of the stem, reporting 99.7% survivorship at five years postoperatively (CA). Stem-related complications were very low, with only three periprosthetic fractures (0.06%), all occurring within one year of implantation. The potential for intraoperative fracture was also extremely low in our study (0.04%) and illuminates the fact that stem insertion via DAA/anterolateral approaches can be very safe using this implant. The cause of femoral stem revision was most often due to infection and none of the stems in this data set were revised for aseptic loosening or subsidence. A significant improvement in the HHS and HOOS scores was noted postoperatively and remained so for the first five years after implantation, representing excellent midterm outcomes.

Aseptic loosening has been described as a failure mechanism of both the femoral and acetabular components. Specifically with regards to the femoral stem, risk factors associated with this include male gender, younger age, and implant design. Bordini et al. found a lower rate of loosening in implants that were coated and anatomical in their fit and had lower rates of subsidence than straight or cemented stems [32]. Stem subsidence is also a concern with uncemented femoral stems. The subsidence rate was found to be higher in stems without a collar as opposed to collared cementless stems at 3.1 mm versus 1.9 mm, respectively, which favors our stem analysis and the low rate of subsidence observed [33]. Loudon and Charnley also found lower rates of subsidence with stems that had a dorsal flange [34]. Finally, femoral stem fractures have also been described as a cause for revision and patient morbidity. Femoral stem fractures were observed more commonly in stems with a proximal buttress in both cemented and cementless stems [35]. The stem in our study had no evidence of fracture and had a robust proximal fit and buttress support, thus lowering this risk.

Thigh pain in our cohort was reported within a range of 3.2% to 8.0% of hips at a minimum of 12 months follow-up. Of those reporting thigh pain, the majority of patients only reported mild-to-moderate symptoms, which were not disabling, and no patients reported marked or disabling thigh pain at four- and five-year follow-ups. This was much lower than the 9% of patients who reported moderate-to-severe thigh pain using a short, cemented stem by Amendola et al. [36]. The incidence of thigh pain after cementless THA was reported by Brown et al. as highly variable, present in 1.9% to 40.4% of cases across a wide range of stem designs [37]. Horwood et al. compared thigh pain with a short stem and reported lower anterior thigh pain than the use of a longer stem at 12% versus 19%, which was higher than our cohort [38]. It is felt that the incidence of thigh pain in our study is within the expected range or better than historical data and not a cause for concern.

Limitations of this study include the design-related shortcoming of being nonrandomized and noncontrolled. Another limitation that might be considered is the variability in the standard-of-care follow-up - not unusual for multicentered, registry-based, patient analysis. This could potentially compromise study cohort compliance. However, the five-year survivorship evaluation of this data set is similar to that reported for the same cup and stem combination in the AJRR, which identified a best-in-class all-cause survival rate of 99.5% at three years. A lack of comparison with a proximally coated, triple-tapered stem without a medial collar does pose as a limitation supporting the role of the collar in the stem, though not yet available. Finally, although our data is very encouraging at five years of follow-up and should predict long-term femoral implant fixation success, further long-term data analysis is needed as relates to this data set and large national registries to further support the value of this implant.

## Conclusions

In this cohort of 5,264 medially collared, hydroxyapatite-coated, triple-tapered stems, the midterm clinical outcomes were excellent. Considering data both from the CA and RA, the stem appears to have a very low complication rate, suggesting safe usage in the early stages of adoption specifically with anterior THA. This stem design is unique and aimed to decrease intraoperative and postoperative complications associated with the anterior approach that were not seen in prior stem designs. The incidence of thigh pain in our study is within the expected range or better than historical data and is not a cause for concern. There were no indications or concerns identified in our study that would prevent its continued use in future applications. This is consistent with the data that show this has the lowest complication rate among other stems in the AJRR database. 
